# ProMod3—A versatile homology modelling toolbox

**DOI:** 10.1371/journal.pcbi.1008667

**Published:** 2021-01-28

**Authors:** Gabriel Studer, Gerardo Tauriello, Stefan Bienert, Marco Biasini, Niklaus Johner, Torsten Schwede

**Affiliations:** 1 Biozentrum, University of Basel, Basel, Switzerland; 2 SIB Swiss Institute of Bioinformatics, Basel, Switzerland; Hebrew University of Jerusalem, ISRAEL

## Abstract

Computational methods for protein structure modelling are routinely used to complement experimental structure determination, thus they help to address a broad spectrum of scientific questions in biomedical research. The most accurate methods today are based on homology modelling, i.e. detecting a homologue to the desired target sequence that can be used as a template for modelling. Here we present a versatile open source homology modelling toolbox as foundation for flexible and computationally efficient modelling workflows. ProMod3 is a fully scriptable software platform that can perform all steps required to generate a protein model by homology. Its modular design aims at fast prototyping of novel algorithms and implementing flexible modelling pipelines. Common modelling tasks, such as loop modelling, sidechain modelling or generating a full protein model by homology, are provided as production ready pipelines, forming the starting point for own developments and enhancements. ProMod3 is the central software component of the widely used SWISS-MODEL web-server.

## Author summary

Knowledge of a protein’s structure permits key insights in its molecular function and supports scientists in the design of targeted and efficient experiments. However, experimental structure determination is a limiting factor and, as a consequence, the number of entries in the Protein Data Bank (PDB) is orders of magnitude lower than the number of known protein sequences. Computational modelling methods can be used to bridge this gap by complementing experimental structure determination. In particular, homology modelling (aka comparative or template-based modelling) approaches which interpolate structural information from homologous structures can provide protein models of sufficiently high accuracy to guide structure based research. In this manuscript we describe ProMod3 which is a modular software package targeted at modelling protein structures by homology. ProMod3 empowers the widely used SWISS-MODEL webserver and already served millions of protein structure models to the scientific community.

This is a *PLOS Computational Biology* Software paper.

## Introduction

The number of entries in the Protein Data Bank (PDB) [1] is orders of magnitude lower than the number of known protein sequences. Computational modelling methods can be used to bridge this gap by complementing experimental structure determination. In particular, homology modelling (aka comparative or template-based modelling) approaches which interpolate structural information from homologous structures can provide protein models of sufficiently high accuracy to guide structure based research [2]. In recent years, robust and scalable pipelines have been developed by the structural bioinformatics community and allow life science researchers to apply these approaches in a fully automated manner at large scale [3,4]. For example, the SWISS-MODEL web-server, developed in our group, is a web based modelling workbench widely used for scientific projects as well as educational purposes [5]. The modelling engine, the software that generates the actual protein model, is a key ingredient of every homology modelling pipeline. Starting from a sequence alignment and template structure(s), it generates coordinates for amino acids aligned to a template scaffold, models regions without template coverage, i.e. insertion-deletion modelling, and constructs sidechain conformations. Additionally, it minimizes the energy of a molecular mechanics force field to resolve stereochemical irregularities and close atomic contacts to finally return a full-atomic model. Various specialized software tools have been developed to perform all or some of these tasks, e.g. MODELLER [6], Rosetta [7], I-Tasser [8], SCWRL [9], or ProModII [10].

SWISS-MODEL is a widely used web service for homology modelling and generates millions of 3D models annually requested by users worldwide. In order to cope with such high workloads, the modelling engine of SWISS-MODEL is required to be computationally efficient, i.e. return a result within minutes, as well as accurate to generate high-quality models suitable for life science applications. To facilitate the development of novel algorithms and implementation of state of the art algorithms, the underlying software framework must be flexible and easily extendible for evolving demands. In order to address these requirements, we have developed ProMod3 as a next generation modelling engine empowering SWISS-MODEL based on the OpenStructure computational structural biology framework [11]. ProMod3 provides efficient data structures which can be manipulated with state-of-the-art algorithms, which can be combined into flexible workflows solving a wide range of modelling problems. The accuracy of models generated by ProMod3 was tested extensively using the Continuous Automated Model EvaluatiOn (CAMEO) platform [12], before ProMod3 has been deployed as default modelling engine in the SWISS-MODEL pipeline as of June 2016, replacing ProModII [10]. Since then, it has served millions of models for the scientific community worldwide. ProMod3 has been implemented *de novo*. Hence ProModII can only be called its predecessor by the naming scheme, without having any common code base.

By making ProMod3 available to the community under the permissive Apache 2.0 open source license, we aim to encourage other groups to adapt the software framework for their specific applications, and to enable open collaboration on future developments of the framework.

Here, we present an overview of the software architecture and implementation, provide benchmarking results on the accuracy and performance of ProMod3, and showcase its application on specific examples.

## Design and implementation

### Software architecture

ProMod3 provides access to common modelling tasks, such as model building, sidechain modelling, etc. by implementing ’actions’ that can be invoked from a command line. For instance, a common starting point is the sequence of a target protein, the structure of a homologue to be used as a template, and an alignment between the template and target sequences. In this simple example, the template structure (e.g. as mmCIF file) and the alignment (e.g. as FASTA file) are passed to ProMod3’s ’build-model’ action which automatically produces a model structure of the target protein (Listing 1).

### Listing 1: Build a homology model from the command line

$ pm build-model -f alignment.fasta -e template.cif

Advanced users benefit from the modular design of ProMod3 that aims at implementing flexible modelling workflows and fast prototyping of novel algorithms using the Python scripting language. For example, the *loop* module provides algorithms and data structures designed to represent, generate and manipulate short peptide segments to model target regions without template information. The *sidechain* module is used to generate all-atom representations of peptide segments. The *scoring* module is concerned with the ranking of alternative conformations and measuring model reliability in general. Higher level modelling tasks utilising the aforementioned modules are gathered in the *modelling* module. Molecular mechanics capabilities to equilibrate structures or segments are available as wrappers for the OpenMM molecular mechanics library [13]. To ensure efficiency, critical algorithms and datastructures have been implemented in C++ and made available to the Python scripting language. This allows for rapid prototyping in Python with the option to port only such components to C++ where computational performance is critical. The following sections summarize the implementation details of the individual ProMod3 modules. Extensive example code can be found in the documentation available at https://openstructure.org/promod3/.

### The *loop* module

ProMod3 mainly relies on OpenStructure (OST) data structures to represent and manipulate molecular structures such as proteins, DNA, RNA and small molecules. ProMod3 complements the OST framework by a BackboneList class that represents a peptide backbone (N, Cα, C, O and Cß atoms). Depending on the use-case, one can access and manipulate Cartesian or internal coordinates which are automatically synchronized. For manipulations in Cartesian space, every atom position has three degrees of freedom that can vary independently from other atoms. Internal coordinates on the other hand use bond lengths, bond angles and dihedral angles to successively define atom positions in a tree-like structure. Assuming constant bond lengths/angles, the internal degrees of freedom can be represented by the dihedral angles. Structural manipulation is simplified as changes introduced by altering a single dihedral angle implicitly propagate through all affected coordinates subsequent in the tree structure. The BackboneList allows for efficient sampling/scoring procedures and can be created from an OST molecular structure. Alternatively, it can be created from scratch by providing a set of dihedral angles or extracted from a database containing structural data. Extraction from a structural database will be discussed in the following sections. Once processing is done, the BackboneList can be inserted back into OST molecular structures.

#### Structural database

The conformational space for short peptide fragments is nowadays largely covered by experimental data [14,15] (S1 Table). The *loop* module implements the StructureDB class to make that data programmatically accessible. The information stored in this database is similar to the Rosetta Vall database [16] and is optimized for fast loading/saving from/to disk, fast access speed and low memory footprint. The following information is stored for every protein chain: amino acid sequence, coordinates of the backbone atoms (N, Cα, C, O), φ/ψ backbone dihedral angles, DSSP [17] secondary structure assignments, solvent accessibilities, residue depths [18], sequence profiles derived from HHblits [19] and sequence profiles derived from structural data [20]. A linear memory layout guarantees fast access to the stored information and allows identifying any fragment in the database by three integer values: the index of the protein chain it belongs to, the offset from the start of that chain and the fragment length. Accessor classes, two of which are described in the following sections, can be built on top of the structural database relating fragments to arbitrary criteria. While examples in the documentation guide through the creation of custom StructureDBs, the StructureDB distributed with ProMod3 contains a non-redundant set of protein chains selected with PISCES [21] using a sequence identity threshold of 60% and a resolution threshold of 2.5 Å. This gives ~21’000 chains with >4’500’000 residues and requires ~500 MB memory.

#### Fragment database

**Fig 1:**
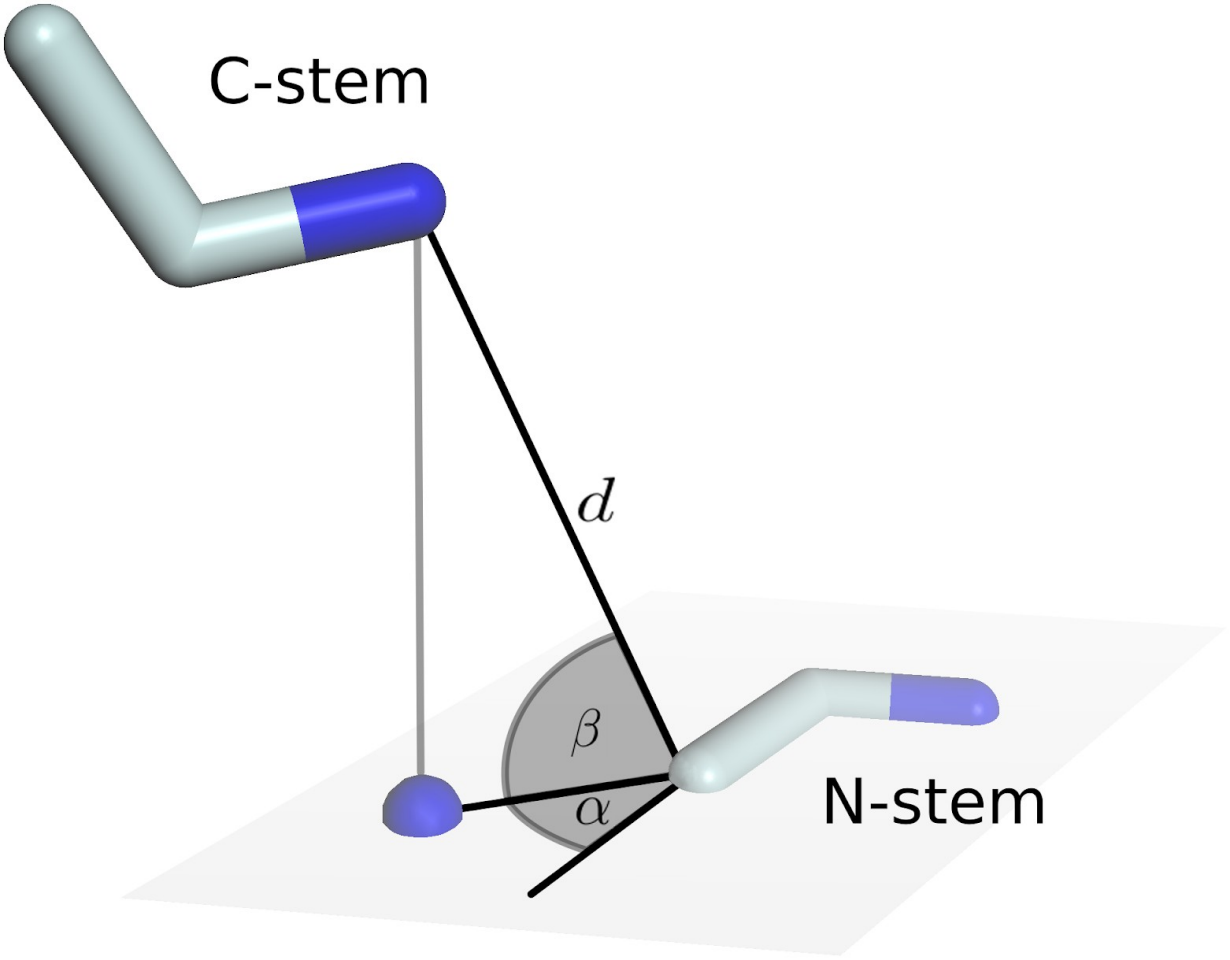
Geometric description of two stem residues. Stem residues are represented by N, Cα and C atoms. Parameters are: number of residues in between (l, not shown), Euclidean distance from N-stem C to C-stem N (d) and four angles. The angles α and β describe the direction towards the C-stem N relative to the N-Stem. Accordingly, the angles γ and δ define the direction towards N-stem C relative to C-stem (not shown).

A typical loop modelling problem involves evaluating multiple loop candidates that are geometrically constrained by two stems [22–24]. The *loop* module implements the FragDB class. This database stores the location of fragments in a specific StructureDB instance and provides access based on geometric criteria. The relative orientation of two stem residues is reduced to six numerical descriptors as visualized in Fig 1. Discretization of the continuous descriptors allows clustering fragments with similar stem geometry. Redundancy in every group is removed by enabling a Cα-RMSD threshold. Fast access to all fragments of such a group is implemented by using the discretized descriptors as key to an internal hash map. The FragDB distributed with ProMod3 stores fragment locations in the default StructureDB. It contains data on ~19’700’000 structurally non-redundant (Cα-RMSD threshold of 1Å) fragments of length 1–12 amino acids with ~3’400’000 distinct stem geometries and requires ~290 MB memory.

#### Fragger

Using short peptide fragments of known protein structures is a common approach to explore the conformational space in ab initio modelling algorithms [25,26]. Suitable fragments can be identified in the StructureDB assuming that sequence based properties exhibit preferences for local structural conformations [27,28]. Fragments sharing similar properties as the desired target sequence thus tend to adequately sample likely conformations. The Fragger class implements a variety of scores to quantify those similarities. They are further described in S1 Text. Due to the linear memory layout of the StructureDB, a sliding window approach allows for an efficient search for a set of fragments optimizing a certain score or a linear combination thereof.

### The *scoring* module

Accurate scoring capabilities to identify near-native conformations are crucial for many modelling tasks such as the selection of alternative local conformations, i.e. selecting one loop candidate among many, guiding sampling procedures or estimating the general local or global reliability of a protein model. Many frequently used scores are physics-based descriptions of macromolecular energy, e.g. AMBER [29], CHARMM [30] or OPLS [31]. Alternatively, the increasing number of experimentally determined protein structures enabled the derivation of knowledge based scores from a statistical perspective. In principle, every structural propensity that can be described probabilistically, e.g. torsion angles or pairwise interatomic distances, can be formulated as statistical potential [32–35] which can be evaluated efficiently. The *scoring* module is concerned with providing a set of scorer classes evaluating individual model properties. Scorer classes implemented in ProMod3 range from stereochemistry related scorers like clash scorers [36] to knowledge based scorers implementing statistical potentials [32]. Additional scorers correlate local structural segments to density information or allow to incorporate arbitrary constraint functions between residue pairs (Cα-Cα or Cβ-Cβ). All available scorers are optimized to efficiently assess local structural stretches given a constant environment and are described in detail in S2 Text. Even though many composite scores exist that simultaneously assess numerous aspects of a protein model by employing multiple scores [37–40], score combination is delegated to the *modelling* module described later, or can be defined by the user. From a design point of view, ProMod3 separates between scorer and environment. Several scorers can be attached to the same environment that contains the actual structural data of the current modelling problem. The environment is updated as modelling proceeds and manages efficient spatial lookups to be used by the attached scorers. Small variations in the protein backbone have a significant impact on sidechain conformation which affects scorer classes that require an all-atom representation. ProMod3 thus allows for efficiently reconstructing the sidechains of segments to be scored as well as residues being close in the environment prior to scoring.

### The *sidechain* module

Due to almost constant bond lengths and bond angles [41], amino acid sidechains can approximately be described with a set of dihedral angles, so called rotamers that cluster around energetically preferred conformations. As a result, libraries have been compiled from structural data that can efficiently reduce the conformational search-space of protein sidechains by proposing rotamers as a starting point for modelling. They either provide rotamers for each amino acid agnostic of the structural context or leverage their dependency on the local backbone configuration [42–47]. ProMod3 provides libraries with and without dependency on the local backbone configuration. All scripts for data extraction and library generation are available to the user as a starting point for custom libraries. Alternatively, the user can read in the backbone dependent Dunbrack 2010 rotamer library [45] provided by the Dunbrack lab. Rotamers can be represented as rigid rotamers (Rigid Rotamer Model, RRM). An alternative are flexible rotamers where the same set of atoms builds the basis for an ensemble of conformations. To better express structural flexibility, these so-called sub-rotamers exhibit small variations around the sidechain dihedral angles (Flexible Rotamer Model, FRM) [48]. For both RRM and FRM, ProMod3 implements the SCWRL4 [9] and SCWRL3 [36] energy functions to estimate pairwise energies between rotamers and towards parts of the protein model that are kept in place. Additionally, ProMod3 implements the VINA energy function [49] which is specifically targeted at evaluating interactions between rotamers and arbitrary chemical compounds. In the case of RRM, all pairwise energies are summed up, whereas FRM exploits a thermodynamics based formalism [48]. Given rotamers and all required pairwise energies, the optimal combination of rotamers that minimizes the overall energy has to be found. This is computationally expensive and a full enumeration of the solution space is not feasible. Preprocessing steps in the form of dead end elimination (DEE) using the Goldstein criterion [50] or edge decomposition [9] are implemented to first reduce the search space. To deterministically identify the global optimum, ProMod3 implements the graph based TreePack algorithm [51,52]. Alternatively, ProMod3 can stochastically sample the search space using a Monte Carlo approach [53,54] or employ the A* algorithm [55] to deterministically identify the global optimum as well as all other solutions within a specified energy threshold.

### The *modelling* module

High level modelling functionality is provided by the modelling module, bringing together the data structures and algorithms of the *loop*, *scoring* and *sidechain* modules. Amongst others, this enables easy access to fitting BackboneLists on stem residues with Cyclic Coordinate Descent (CCD, [56]) /KInematic Closure (KIC, [57]), loop modelling by Monte Carlo algorithms and interfaces to the molecular mechanics functionality in OST for relaxation/minimization. The modelling module also implements a complete modelling application, which will be described in more detail, including key pipelines used therein for loop modelling and sidechain modelling. The pipelines are designed to be computationally efficient and flexibly extendable while comparing favorably when benchmarked against commonly used tools.

#### Homology modelling pipeline

The default homology modelling pipeline is designed as a compromise between speed and accuracy as required for applications like SWISS-MODEL. Every step of the pipeline can be customized as later demonstrated in the results section. Given an alignment and a template structure, conserved structural information is transferred to construct an initial model exhibiting the desired target sequence. Small deletions are processed by relaxing neighbouring residues and resolved if a stereochemically valid conformation can be obtained. Non-resolved deletions from now on get treated the same way as insertions, processed successively by the default loop modelling pipeline. Once the model has a continuous backbone, sidechains are reconstructed using the default sidechain modelling pipeline. Energy minimization resolves stereochemical irregularities and clashes introduced in the modelling process. Short steepest descent and conjugate gradient minimization runs are iteratively applied to the model until all stereochemical problems are resolved or a maximum number of iterations is reached.

#### Loop modelling pipeline

The loop modelling pipeline aims at modelling stereochemically realistic loops that are anchored by two flanking stem residues. It primarily relies on the StructureDB/FragDB databases from the *loop* module to (1) propose loop candidates and (2) select one of them by employing the *scoring* module. Given the observed structural coverage in the default StructureDB (S1 Table), loop candidates that sufficiently sample the accessible conformational space can be expected up to a loop length of around 12 residues. In case of longer loops or no success with the database approach, a Monte Carlo sampling procedure (3) is used as fallback.

**(1) Proposing loop candidates**: The default StructureDB/FragDB databases are queried using the initial stem residues as anchor. If no or not enough stereochemically realistic loop candidates can be identified, the stems are shifted, thus elongating the initial loop up to a maximum length of 12 residues. Shifting is performed by the ScoringGapExtender class that returns an ordered list of possible elongations to sequentially query the databases until enough loop candidates are found. The ordering aims at first processing elongations that are likely to omit structurally less conserved residues from the template. The found loop candidates are fitted to their respective stems using CCD before scoring.

**(2) Scoring and candidate selection**: As a compromise between speed and accuracy, the default loop modelling pipeline primarily relies on backbone related scores (CBPackingScore, CBetaScore, ClashScore, HBondScore, ReducedScore, TorsionScore, see S2 Text for details). They are complemented by the RMSD of the loop stems before applying CCD as well as database specific scores that compare the sequence/structure profiles from the loop candidate (stored in StructureDB) with the target sequence profile. The scores are linearly combined to select a final candidate (see S3 Text for estimation of linear weights). The application of scores involving a full atomic model (AllAtomInteractionScore, AllAtomPackingScore, AllAtomClashScore) can be enabled at the cost of increased runtime, as the sidechains for each loop candidate as well as the sidechains in close proximity need to be (re-)modelled individually.

**(3) Monte Carlo sampling**: The accessible conformational space between the stem residues is explored using a simulated annealing scheme. Backbone dihedral angles of randomly selected loop residues are altered by drawing from probability distributions used for the corresponding Fragger score (S1 Text). The elongation scheme described in (1) but without restrictions on loop length is applied if no stereochemically realistic loops can be generated. Sampling based on structural fragments can be enabled but increases runtime as they first need to be derived using the Fragger.

#### Sidechain modelling pipeline

The ProMod3 default sidechain modelling pipeline follows the same steps as SCWRL4, except for one additional post-processing step: sub-rotamer optimization. Rotamers (Flexible Rotamer Model—FRM) are extracted from our internal backbone dependent rotamer library for all residues with incomplete sidechains. Backbone atoms as well as sidechain atoms from complete residues are fixed, meaning they contribute to the energy evaluation but remain in place. Furthermore, cysteines that are able to build disulfide bonds given the extracted rotamers are detected and fixed. Upon energy calculation with the SCWRL4 energy function, complexity reduction with DEE and edge decomposition, the optimal configuration of rotamers is determined using TreePack. Every rotamer in the FRM is represented by an ensemble of sub-rotamers. The central sub-rotamer could be considered the representative of the ensemble and applied to the input structure. Instead, a sub-rotamer optimization is performed. Every rotamer that is part of the solution is transformed to a set of rigid rotamers representing all of its sub-rotamers. Those sets re-enter optimization to decide on the optimal sub-rotamers which are then applied to the input structure. This final step aims to reduce clashes with limited computational cost by sampling the structural flexibility of the FRM. Similar approaches, e.g. CIS-RR [58] or RASP [59], refine their initial solutions with explicit rotamer relaxation but report considerable computational overhead in the relaxation step.

## Results

The default implementations for loop and sidechain modelling are benchmarked versus commonly used methods on external test sets consisting of experimentally determined structures. The full homology modelling workflow is assessed on real world homology modelling problems and directly compared to the MODELLER [6] modelling engine. In the following section we provide the benchmarking results and describe a customized homology modelling pipeline to exemplify the use of the previously described ProMod3 modules. All data and scripts to reproduce the presented results are available at https://git.scicore.unibas.ch/schwede/promod3_pipeline_benchmark.

### Loop modelling accuracy

Accuracy of the default loop modelling pipeline has been evaluated on a benchmark data set defined in [22]. It consists of 510 loops in high resolution X-ray structures, 30 for every loop length within four and twenty residues. In the aforementioned work, this benchmark set has been used to assess the commonly used loop modelling algorithms MODELLER [6], Rapper [60,61], PLOP [62] and original FREAD [63]. Here we complement this assessment with the default loop modelling pipeline in ProMod3. To avoid redundancy, the ProMod3 pipeline for this benchmark used a StructureDB/FragDB with no entry exhibiting a sequence identity > 90% to any of the protein chains from the benchmark set. ProMod3 performs well for shorter loops that predominantly need to be modelled in the CAMEO homology modelling benchmark which we believe to largely cover realistic homology modelling scenarios (Table 1 and S1 Fig). Longer loops, in particular loops longer than 12 residues that are modelled with the Monte Carlo fallback, exhibit a decline in modelling accuracy. Rapper and original FREAD show numerically better results in these cases. There are methods that report sub-angstrom accuracy for loop lengths around 12 residues (e.g. GalaxyLoop [64], Rosetta-NGK [65], Sphinx [66]) but the improvements come with significant computational costs. ProMod3 generates results in a few seconds for the database approach on common computer hardware. Also the Monte Carlo fallback typically requires well below one minute per loop modelling problem.

**Table 1:** Loop modelling benchmark.

Length	MODELLER	Rapper	PLOP	Orig. FREAD	ProMod3
4	1.73	1.10	1.79	1.29	0.61
5	2.30	1.23	2.76	2.19	0.63
6	2.38	1.92	3.25	1.79	1.02
7	3.44	2.60	3.73	2.53	1.32
8	4.25	2.88	4.34	2.88	2.14
9	4.31	3.03	5.58	3.08	2.10
10	5.69	3.90	6.41	4.25	2.98
11	5.34	4.63	6.52	4.55	2.84
12	7.18	5.10	6.86	3.99	3.84
13	6.96	5.72	7.86	5.54	6.67
14	7.24	6.02	8.37	6.07	7.57
15	7.93	6.41	9.60	6.41	7.56
16	8.65	7.29	9.86	7.50	8.12
17	9.61	7.35	9.00	7.84	9.74
18	7.64	7.56	10.54	5.48	10.85
19	10.52	9.10	11.51	7.67	9.31
20	10.49	10.64	11.14	7.64	11.76

Average backbone RMSD in Å (N, Cα, C, O) for different loop lengths in the FREAD benchmark set. ProMod3 results complement the data extracted from Table I in the FREAD manuscript [22].

### Sidechain modelling accuracy

**Fig 2:**
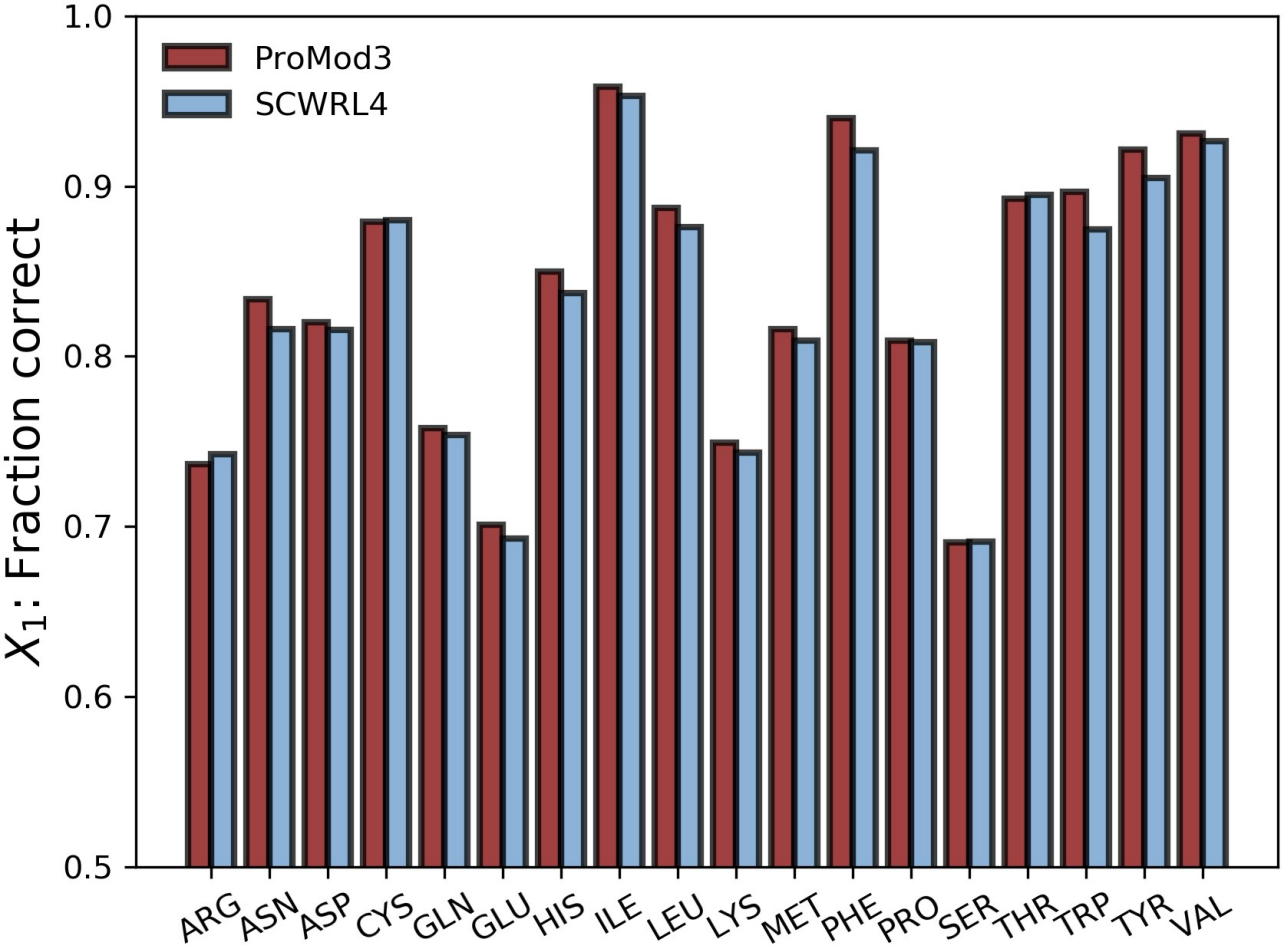
Sidechain modelling benchmark. Comparison of ProMod3 sidechain modelling performance with SCWRL4 by measuring the fraction of χ_1_ angles being within 20° of the reference angles observed in the SCWRL4 test set.

The test set described in the SCWRL4 manuscript has been used to evaluate sidechain modelling accuracy. It consists of 379 experimentally determined protein structures. All sidechains of the crystallographic asymmetric units have been reconstructed with the default sidechain modelling pipeline as well as SCWRL4. In case of multiple chains with the same sequence, only the first chain is considered for evaluation. The fraction of χ_1_ angles within 20° of the reference value in the crystal structure, a widely used measure in the field [9,36,54,67], is used as the main criteria for reconstruction accuracy. Additionally, the effectiveness of the sub-rotamer optimization is measured by the number of clashing sidechains, i.e. sidechains having at least one atom closer than 0.6*σ to any other atom from a different residue. σ is derived from the Lennard-Jones parameterization of the respective atom types in CHARMM22 [68]. The algorithmic similarity in ProMod3 and SCWRL4 leads to comparable accuracy in sidechain modelling (avg. fraction of correct χ_1_: 83.31% (ProMod3), 82.67% respectively). On a per amino acid basis, the ProMod3 improvements for bulky sidechains (PHE: 94.00% vs. 92.08%, TYR: 92.16% vs. 90.49%, TRP: 89.68% vs. 87.44%) are more prominent (Fig 2 and S2 Table). The sub-rotamer optimization not only largely resolves clashing sidechains (54 with optimization vs. 543 without, SCWRL4: 556) but also improves the sidechain orientation in those cases (S2 Table). Even with sub-rotamer optimization, a speedup of 3.0x (2.2x when not using sub-rotamers at all (RRM) in both, ProMod3 and SCWRL4) compared to SCWRL4 can be observed on that test set with timing details available in S4 Text.

### Homology modelling accuracy

**Fig 3:**
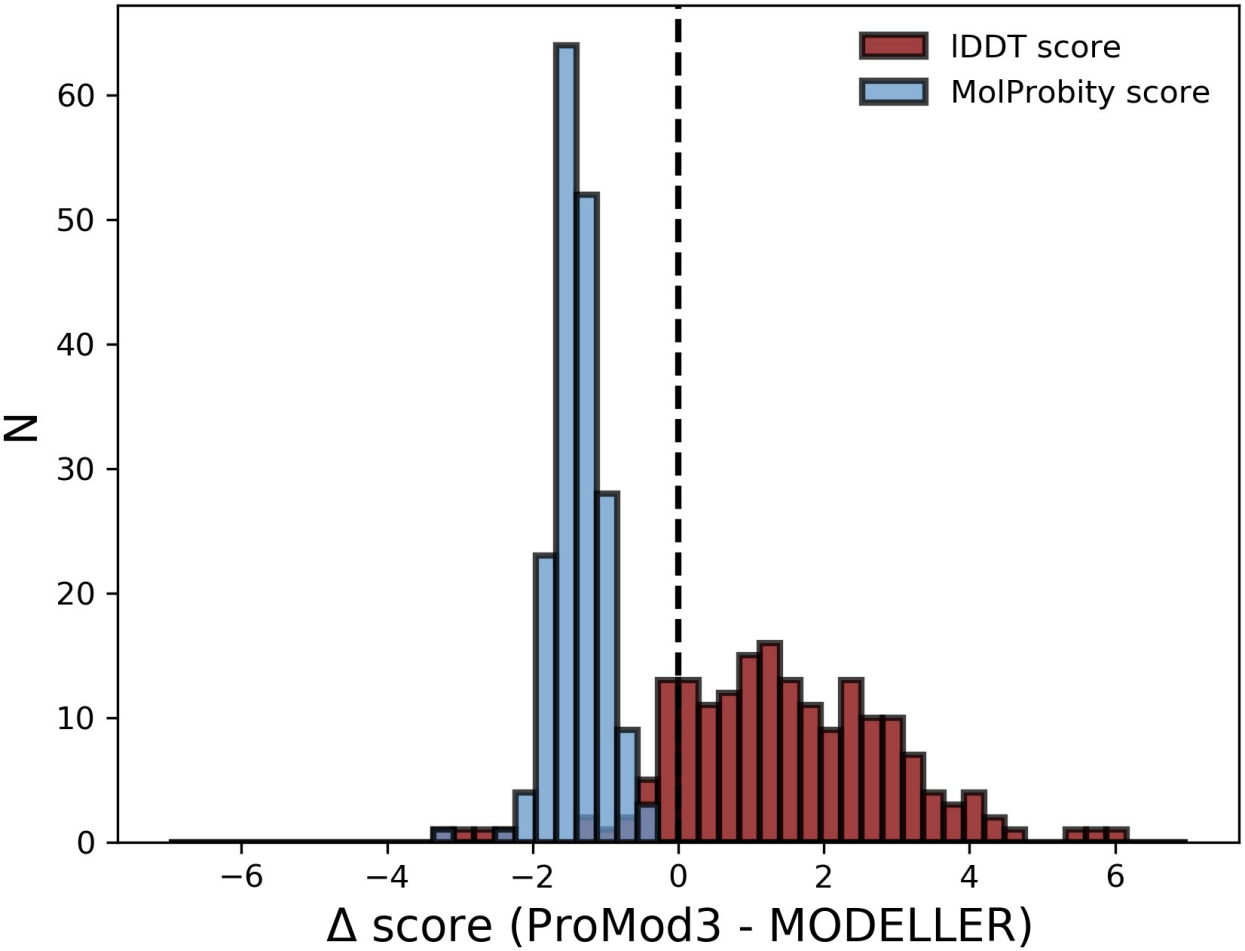
Homology modelling benchmark. Difference of homology modelling performance between ProMod3 and MODELLER. The same data serves as input for both engines to create models for 190 target sequences. The similarity to the native structure is measured by the lDDT score (red, higher is better) and stereochemistry by the MolProbity score (blue, lower is better).

The 190 target sequences submitted by the 3D category of CAMEO during the time range 2020.03.28–2020.06.13 are used as a homology modelling accuracy benchmark. A profile-profile-based HHblits template search on the SWISS-MODEL template library (SMTL) [5] has been performed for each target sequence on the day of the CAMEO submission before the according target structure was released to the public. Always selecting the template with the best HHblits e-value allows to run ProMod3 (version 3.1.0) and MODELLER (version 9.24) with default settings given the same input data (template structure, target-template sequence alignment, HHblits sequence profile). Before evaluation, terminal extensions not covered by the provided template structure were removed since such extensions in a model are likely to be of low quality without extensive *de novo* modelling efforts and therefore not within scope for this homology modelling benchmark. Modelling accuracy is measured by the superposition-free all-atom-based lDDT score [69] which quantifies the consistency of interatomic distances in native structure and model in a range of [0, 100]. The overall MolProbity score [70] evaluates stereochemistry as an additional but equally important aspect. ProMod3 shows an average increase in lDDT score of 1.51 (Fig 3, see S2 Fig for a per-model analysis). Regarding overall MolProbity score, ProMod3 shows an average decrease of 1.37 (Fig 3, see S2 Fig for a per-model analysis). The overall MolProbity score is intended to relate with X-ray resolution, lower is therefore better. The decomposition into its single components (clashscore, Ramachandran outliers and rotamer outliers) identifies clashes as the main cause for the observed discrepancy (S2 Fig). While building better models with improved stereochemistry, ProMod3 builds the models of the test set moderately faster by a factor of 1.3 with timing details available in S4 Text. An equivalent comparison with ProModII (version 3.70) is available in S3 Fig.

### Example modification of a modelling pipeline

**Fig 4:**
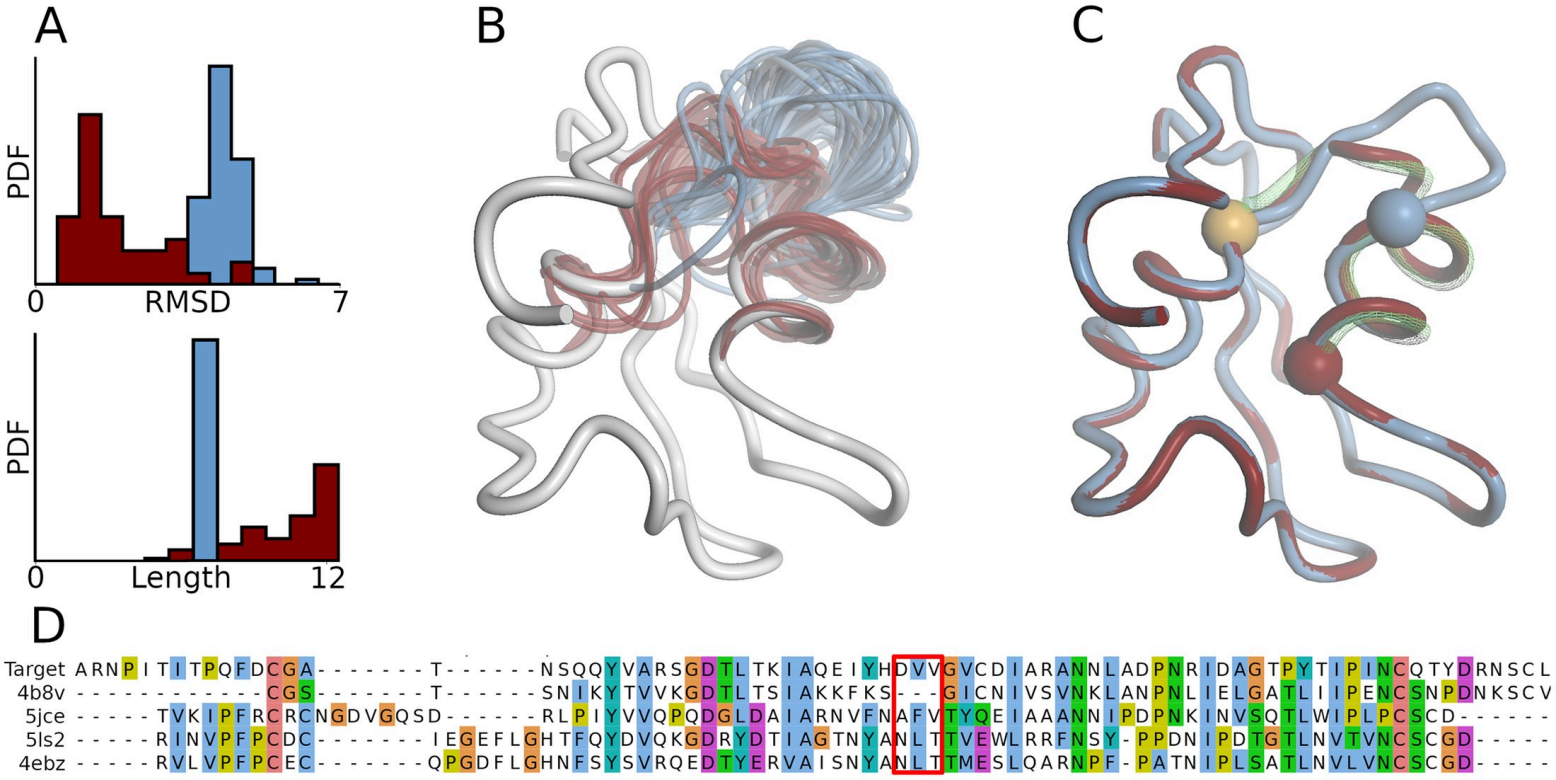
Custom modelling pipeline. (A) Probability densities for backbone RMSD (top) /length (bottom) of loop candidates processed in default pipeline (blue, N = 83) and custom pipeline (red, N = 40). (B) Loop candidates from A superposed onto optimal template structure (white, PDB ID: 4B8V). (C) Model built with default (blue) and custom pipeline (red). Spheres mark the stem residues flanking the inserted loops. Both models share the same C-stem (orange) but the N-stems differ. The green wireframe represents the loop from the target structure (PDB ID: 6Q40). (D) Sequence alignment with problematic insertion marked red. The first two sequences represent the target and globally optimal template. The last three represent templates that are globally suboptimal but do not contain the problematic insertion.

To showcase how easy it is to modify the default modelling pipeline for a specific problem, we look at the structure of a LysM domain-containing protein from *zymoseptoria tritici* (UniProtKB: F9XHX3) that has been released in the protein data bank on October 16 2019 (PDB ID: 6Q40). Prior to release, CAMEO sent the sequence as a modelling target to registered modelling servers (CAMEO target ID: 2019-10-12_00000117_1). Among 61 found templates, SWISS-MODEL correctly identified a homologue from *passalora fulva* (PDB ID: 4B8V) as optimal template for the default modelling pipeline (global lDDT: 62.04). However, an insertion between H38 and G42 is modelled suboptimally even though this loop participates in a functionally relevant dimerization interface [71] (Fig 4C and 4D). The idea pursued by this section is to exemplify a customization of the default loop modelling pipeline in ProMod3. In the first step, all 61 templates found by SWISS-MODEL are used to build a custom StructureDB and FragDB (Listing 2). Secondly one iteration of loop modelling is performed with the custom databases in order to introduce a bias towards loop candidates that are homologous to the target structure. The default modelling pipeline is then invoked to model remaining gaps, sidechains and minimize model energy (Listing 3). The model generated by the custom pipeline accurately models the problematic insertion which results in an increase of global lDDT score from 62.04 to 69.63. Due to the high structural coverage of the default StructureDB, the default loop modelling pipeline already identifies stereochemically valid loop candidates with few elongation steps and proceeds to candidate selection. However, none of the candidates is close to native (Fig 4A and 4B). The custom databases on the other hand only contain structural data from homologues, three of which not containing the problematic insertion. 35 of the 40 processed loop candidates originate from those three. Even though those loop candidates are longer than the ones extracted from the default databases, they are more accurate (Fig 4A and 4B) and the selection procedure successfully closes the insertion with sub-angstrom accuracy (Fig 4C).

### Listing 2: Create custom StructureDB/FragDB

import pandas as pd

from ost import io

from promod3 import loop, modelling

# Load csv file with file paths etc. of found templates

data_table = pd.read_csv(’data.csv’)

# Create StructureDB and fill with prepared data

struct_db = loop.StructureDB(loop.StructureDBDataType.Minimal)

for tpl in data_table.itertuples():

  coords = io.LoadPDB(tpl.pdb_file)

  seqres = io.LoadSequence(tpl.seqres_file)

  struct_db.AddCoordinates(tpl.pdb_id, tpl.chain_name,

   coords, seqres)

# Create FragDB which refers to our StructureDB

frag_db = loop.FragDB(1.0, 20)

for fragment_length in range(3, 15):

  frag_db.AddFragments(fragment_length, 1.0, struct_db)

# Save databases

frag_db.Save(’frag_db.dat’)

struct_db.Save(’struct_db.dat’)

### Listing 3: Custom modelling pipeline

from ost import io, mol

from promod3 import loop, modelling

# Load template and alignment

tpl = io.LoadPDB(’data/4b8v_A_BLAST.pdb’)

mol.alg.AssignSecStruct(tpl)

aln = io.LoadAlignment(’data/4b8v_A_BLAST_aln.fasta’)

aln.AttachView(1, tpl.CreateFullView())

# Modelling algorithms operate on a modelling handle

# Besides coordinates it tracks non-closed gaps etc.

mhandle = modelling.BuildRawModel(aln)

# Load custom databases and try to close gaps in mhandle

frag_db = loop.FragDB.Load(’frag_db.dat’)

struct_db = loop.StructureDB.Load(’struct_db.dat’)

modelling.FillLoopsByDatabase(mhandle, frag_db, struct_db)

# Invoke default modelling pipeline to model remaining gaps,

# sidechains and minimize model energy

final_model = modelling.BuildFromRawModel(mhandle)

io.SavePDB(final_model, ’model.pdb’)

### Availability and future directions

The source code of ProMod3 is available at https://git.scicore.unibas.ch/schwede/ProMod3. The data underlying the results presented in this study are available at https://git.scicore.unibas.ch/schwede/promod3_pipeline_benchmark. Extensive documentation is hosted on https://openstructure.org/promod3. A rich set of example code, including the required input to run it, helps new users to get started. All examples and a large part of the source code are evaluated by a unit testing framework to ensure production ready software quality. Future developments will be tightly coupled with new developments of the SWISS-MODEL web service which also guarantees active maintenance. Furthermore, we envision open collaborations with other groups to introduce new features and functionality. The developers of the project can be approached using the OpenStructure user mailing list (openstructure-users@maillist.unibas.ch).

## Supporting information

S1 Fig Length of modelled loops in homology modelling benchmark. Length of all 937 loop modelling problems resolved by the default loop modelling pipeline in the CAMEO based homology modelling benchmark. Initial length is given by the input alignment, whereas resolved length corresponds to the actually modelled loop after potential elongation as described for the default loop modelling pipeline. An initial length of 0 indicates a deletion. 95.9% of all resolved lengths are equal or less than 12 (illustrated by vertical line) and therefore a product of the database approach. The remnant is modelled using the Monte Carlo fallback. 6 initial and 7 resolved stretches are longer than 25 residues and not shown in the histogram (see data availability statement for raw data access).
(TIF)

S2 Fig ProMod3/MODELLER per-model comparison in homology modelling benchmark. Models for each target in the homology modelling benchmark are built with ProMod3/MODELLER (default settings) using the same input data. Every dot represents two models of the same target. Average lDDT scores (a) for ProMod3/MODELLER: 59.04/57.54, average MolProbity overall scores (b): 1.70/3.07, average MolProbity clash scores (c): 5.18/85.31, average MolProbity rotamer outliers (d): 1.61/3.30 and average MolProbity Ramachandran outliers (e): 1.64/1.59.
(TIF)

S3 Fig ProMod3/ProModII comparison in homology modelling benchmark. Models for each target in the homology modelling benchmark are built with ProMod3/ProModII (default settings) using the same input data. The evaluation has been performed on a subset of 169 models for which ProModII successfully delivered a result. (a) Equivalent of Fig 3. Remaining subplots are the equivalent of S2 Fig. Average lDDT scores (b) for ProMod3/ProModII: 60.26.04/57.58, average MolProbity overall scores (c): 1.67/2.92, average MolProbity clash scores (d): 5.12/96.74, average MolProbity rotamer outliers (e): 1.49/1.56 and average MolProbity Ramachandran outliers (f): 1.60/3.04.
(TIF)

S1 Table Structural coverage in default StructureDB. For every length between 3 and 15, a subset containing 1000 fragments has randomly been selected from all possible fragments in the default StructureDB. “Fraction Covered” reports the fraction of that subset for which a fragment from another entry in the default StructureDB with Cα-RMSD < 1Å can be found (average over 3 runs). “Fraction Covered (Coil)” reports the same number but the random subsets only consist of fragments with at least 50% of the residues being assigned as coil by DSSP.
(PDF)

S2 Table Sidechain modelling accuracy raw data. Comparison of ProMod3 sidechain modelling performance (a with sub-rotamer optimization, b without sub-rotamer optimization) with SCWRL4 on a test set described in the SCWRL4 manuscript. Reported are the fraction of χ_1_ angles being within 20° of the reference angles, the average RMSD in Å of sidechain atoms (not including Cβ) and the number of sidechains involved in clashes.
(PDF)

S1 Text Fragger scores. (PDF)

S2 Text Scorers of the *scoring* module. (PDF)

S3 Text Linear weights for loop candidate selection. (PDF)

S4 Text Speed benchmarks. (PDF)
