# Supplementary figures and images for: ProMod3—A versatile homology modelling toolbox
Source: PLoS Comput Biol. 2021 Jan 28;17(1):e1008667. doi: 10.1371/journal.pcbi.1008667 (PMC7872268; doi:10.1371/journal.pcbi.1008667)

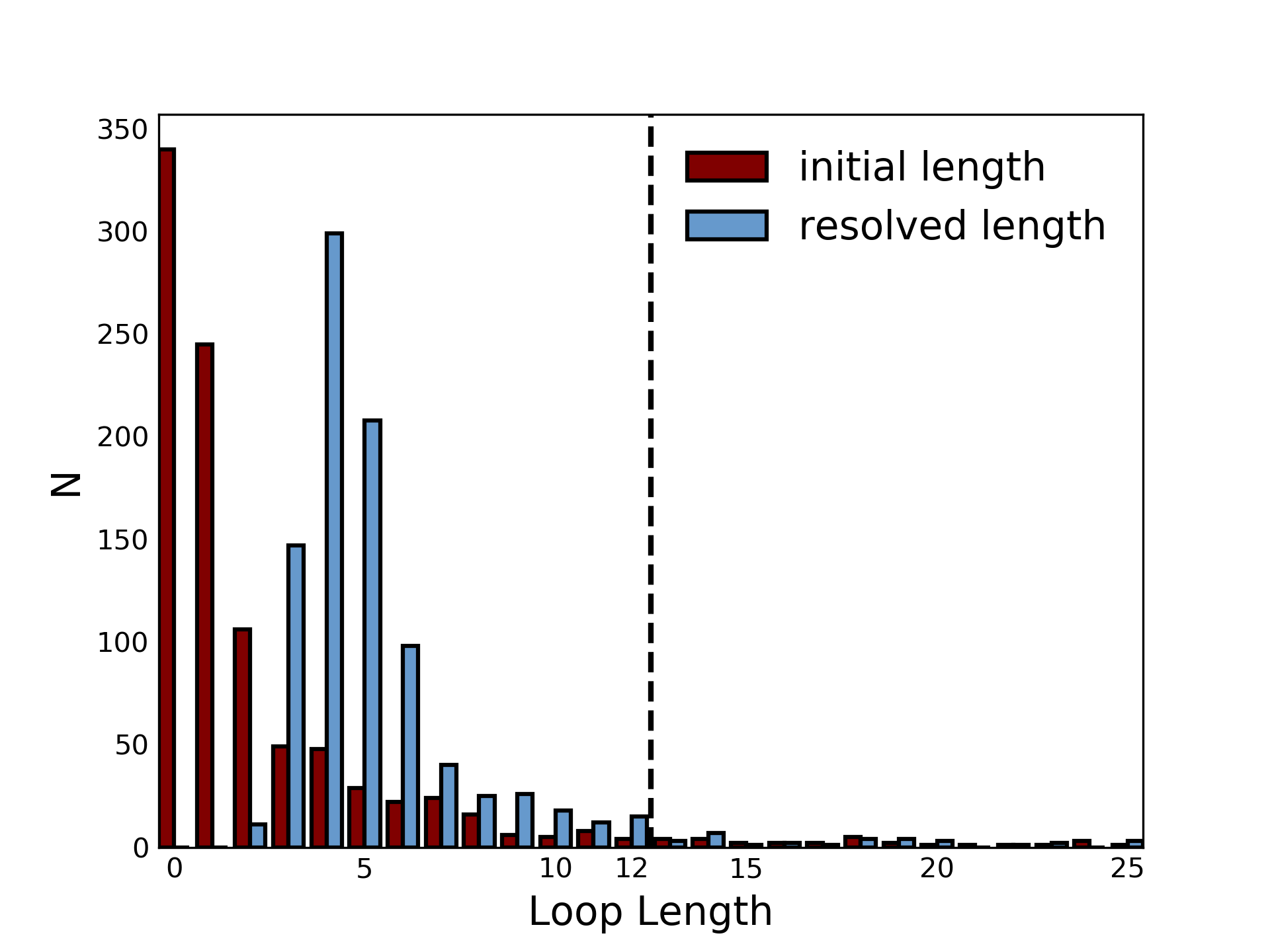

Supplement: S1 Fig — Length of all 937 loop modelling problems resolved by the default loop modelling pipeline in the CAMEO based homology modelling benchmark. Initial length is given by the input alignment, whereas resolved length corresponds to the actually modelled loop after potential elongation as described for the default loop modelling pipeline. An initial length of 0 indicates a deletion. 95.9% of all resolved lengths are equal or less than 12 (illustrated by vertical line) and therefore a product of the database approach. The remnant is modelled using the Monte Carlo fallback. 6 initial and 7 resolved stretches are longer than 25 residues and not shown in the histogram (see data availability statement for raw data access). (TIF) [file pcbi.1008667.s001.tif]

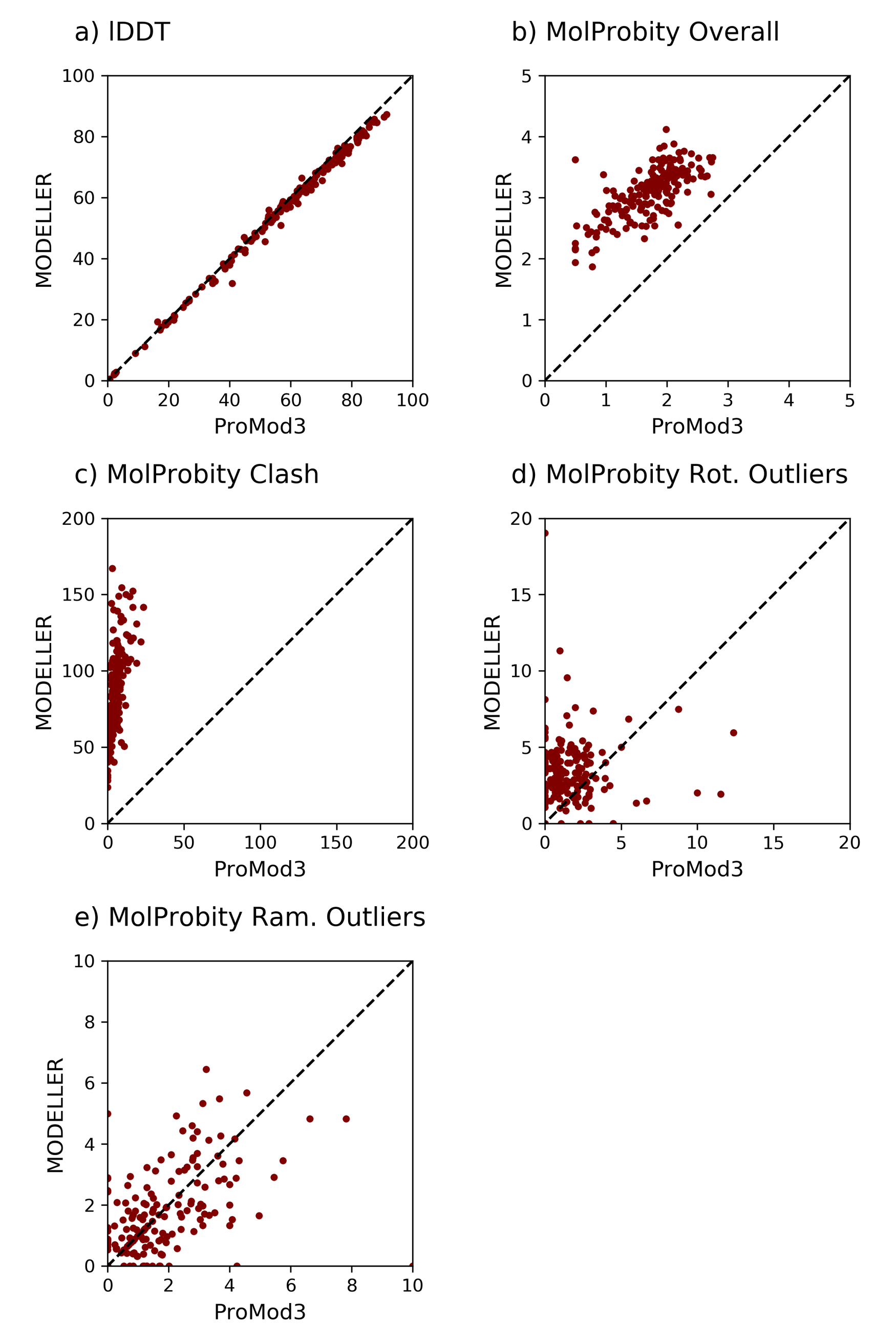

Supplement: S2 Fig — Models for each target in the homology modelling benchmark are built with ProMod3/MODELLER (default settings) using the same input data. Every dot represents two models of the same target. Average lDDT scores (a) for ProMod3/MODELLER: 59.04/57.54, average MolProbity overall scores (b): 1.70/3.07, average MolProbity clash scores (c): 5.18/85.31, average MolProbity rotamer outliers (d): 1.61/3.30 and average MolProbity Ramachandran outliers (e): 1.64/1.59. (TIF) [file pcbi.1008667.s002.tif]

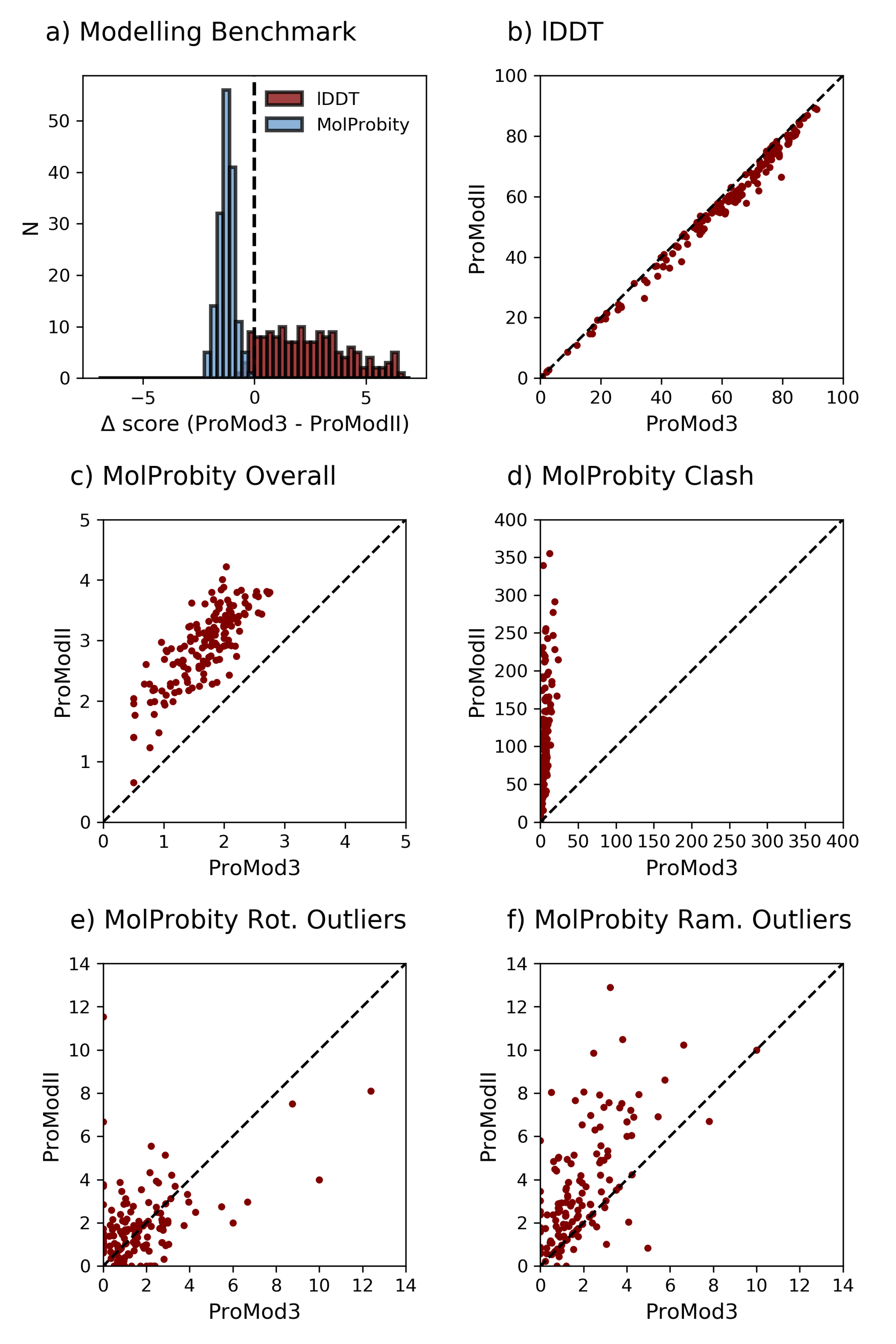

Supplement: S3 Fig — Models for each target in the homology modelling benchmark are built with ProMod3/ProModII (default settings) using the same input data. The evaluation has been performed on a subset of 169 models for which ProModII successfully delivered a result. (a) Equivalent of Fig 3. Remaining subplots are the equivalent of S2 Fig. Average lDDT scores (b) for ProMod3/ProModII: 60.26.04/57.58, average MolProbity overall scores (c): 1.67/2.92, average MolProbity clash scores (d): 5.12/96.74, average MolProbity rotamer outliers (e): 1.49/1.56 and average MolProbity Ramachandran outliers (f): 1.60/3.04. (TIF) [file pcbi.1008667.s003.tif]
