# Supplementary material for: ProMod3—A versatile homology modelling toolbox
Source: PLoS Comput Biol. 2021 Jan 28;17(1):e1008667. doi: 10.1371/journal.pcbi.1008667 (PMC7872268; doi:10.1371/journal.pcbi.1008667)
Supplement: S1 Table — For every length between 3 and 15, a subset containing 1000 fragments has randomly been selected from all possible fragments in the default StructureDB. “Fraction Covered” reports the fraction of that subset for which a fragment from another entry in the default StructureDB with Cα-RMSD < 1Å can be found (average over 3 runs). “Fraction Covered (Coil)” reports the same number but the random subsets only consist of fragments with at least 50% of the residues being assigned as coil by DSSP. (PDF) [file pcbi.1008667.s004.pdf]

Length	Fraction Covered	Fraction Covered (Coil)
3	1.000	1.000
4	1.000	1.000
5	1.000	1.000
6	1.000	1.000
7	1.000	1.000
8	1.000	1.000
9	1.000	1.000
10	0.995	0.984
11	0.981	0.907
12	0.937	0.791
13	0.905	0.592
14	0.847	0.500
15	0.809	0.366
