# Supplementary material for: ProMod3—A versatile homology modelling toolbox
Source: PLoS Comput Biol. 2021 Jan 28;17(1):e1008667. doi: 10.1371/journal.pcbi.1008667 (PMC7872268; doi:10.1371/journal.pcbi.1008667)
Supplement: S2 Table — Comparison of ProMod3 sidechain modelling performance (a with sub-rotamer optimization, b without sub-rotamer optimization) with SCWRL4 on a test set described in the SCWRL4 manuscript. Reported are the fraction of χ1 angles being within 20° of the reference angles, the average RMSD in Å of sidechain atoms (not including Cβ) and the number of sidechains involved in clashes. (PDF) [file pcbi.1008667.s005.pdf]

		X_1 correct (%)			Avg. RMSD			N Clashing Residues		
AA	N	ProMod3[a]	ProMod3[b]	SCWRL4	ProMod3[a]	ProMod3[b]	SCWRL4	ProMod3[a]	ProMod3[b]	SCWRL4
ARG	3601	73.67	73.76	74.23	1.96	1.97	1.93	6	156	159
ASN	2875	83.34	83.17	81.60	0.63	0.63	0.67	1	9	7
ASP	4012	82.01	82.28	81.56	0.77	0.77	0.82	3	38	34
CYS	999	87.89	87.69	87.99	0.18	0.18	0.20	2	2	1
GLN	2501	75.77	76.33	75.37	1.11	1.11	1.13	1	15	18
GLU	4611	70.11	70.07	69.29	1.49	1.48	1.49	0	83	90
HIS	1542	84.95	85.02	83.72	0.89	0.89	0.95	2	17	25
ILE	3964	95.86	95.71	95.31	0.26	0.26	0.27	3	13	9
LEU	6554	88.74	88.59	87.60	0.33	0.33	0.36	6	13	21
LYS	3819	74.94	75.18	74.31	1.12	1.13	1.14	0	15	18
MET	1406	81.58	81.93	80.94	0.74	0.75	0.78	0	4	1
PHE	2715	94.00	92.04	92.08	0.61	0.67	0.72	7	18	22
PRO	3230	80.93	80.93	80.80	0.14	0.14	0.14	4	3	6
SER	4101	69.06	69.03	69.11	0.32	0.32	0.33	1	6	5
THR	3784	89.27	89.19	89.48	0.21	0.21	0.21	1	4	11
TRP	979	89.68	87.54	87.44	0.99	1.07	1.10	3	25	21
TYR	2346	92.16	90.58	90.49	0.74	0.80	0.86	13	118	104
VAL	5018	93.08	93.20	92.63	0.25	0.24	0.26	1	4	4
