# Supplementary material for: ProMod3—A versatile homology modelling toolbox
Source: PLoS Comput Biol. 2021 Jan 28;17(1):e1008667. doi: 10.1371/journal.pcbi.1008667 (PMC7872268; doi:10.1371/journal.pcbi.1008667)
Supplement: S1 Text — (PDF) [file pcbi.1008667.s006.pdf]

Fragger scores

SeqID: Fraction of identical amino acids to the query sequence.
SeqSim: Avg. substitution score to the target sequence as estimated by any substitution matrix available in OST, e.g. BLOSUM62.
TorsionProbability: Avg. probabilities of *φ/ψ* backbone dihedral angles in the structural database given the input sequence. The probability distribution to score one particular location in the structural database is selected based on the identity of the central residue and the identity of the two flanking residues. Instead of generating probability distributions for all possible combinations of amino acid triplets, the amino acids can be grouped arbitrarily. The default grouping scheme follows Solis & Rachovsky [1]. Default distributions are available to the user but custom distributions can be generated with custom grouping schemes and training data.
SSAgreement: Avg. secondary structure agreement score given a PSIPRED [2] prediction and the observed secondary structure in the structural database as estimated by DSSP [2,3]. The used formalism is probabilistic [4]:

$$S(d,p,c) = log\left(\frac{p(d,p,c)}{p(d)p(p,c)}\right)$$

where *d* is the secondary structure assignment by DSSP, *p* the secondary structure prediction of the target sequence from PSIPRED and *c* the according PSIPRED confidence value. The underlying probability distributions have been generated based on a non-redundant set of experimentally determined protein structures.
SeqProfile: Avg. L1 distance of profile columns in a target sequence profile and the sequence profiles present in the structural database. The same formalism is used in the Rosetta fragment picking protocol [5]:

$$S(p,q) = \sum_{i=1}^{20} |p(i) - q(i)|$$

where *p(i)* represent the probabilities of the 20 proteinogenic amino acids in the target sequence profile and *q(i)* the same in the sequence profile stored in the structural database. An alternative formalism as it is in use in HHsearch would be [4]:

$$S(p,q) = log\left(\sum_{i=1}^{20} \frac{p(i)q(i)}{f(i)}\right)$$

where *f* additionally represents a reference distribution. This is computationally more expensive but did not improve performance in fragment detection (data not shown).
StructProfile: Same as SeqProfile but the target profile is compared to the structural profiles in the structural database.

References

1. Solis AD, Rackovsky S. Improvement of statistical potentials and threading score functions using information maximization. Proteins. 2006;62: 892–908.

2. Jones DT. Protein secondary structure prediction based on position-specific scoring matrices 1 1Edited by G. Von Heijne [Internet]. Journal of Molecular Biology. 1999. pp. 195–202. doi:10.1006/jmbi.1999.3091

3. Kabsch W, Sander C. Dictionary of protein secondary structure: pattern recognition of hydrogen-bonded and geometrical features. Biopolymers. 1983;22: 2577–2637.

4. Söding J. Protein homology detection by HMM-HMM comparison. Bioinformatics. 2005;21: 951–960.

5. Gront D, Kulp DW, Vernon RM, Strauss CEM, Baker D. Generalized fragment picking in Rosetta: design, protocols and applications. PLoS One. 2011;6: e23294.
