# Supplementary material for: ProMod3—A versatile homology modelling toolbox
Source: PLoS Comput Biol. 2021 Jan 28;17(1):e1008667. doi: 10.1371/journal.pcbi.1008667 (PMC7872268; doi:10.1371/journal.pcbi.1008667)
Supplement: S2 Text — (PDF) [file pcbi.1008667.s007.pdf]

Scorers of the *scoring* module

CBPackingScore: Statistical potential that evaluates the number *N* of other Cβ positions within a *cutoff* of the Cβ position of the residue to be evaluated.

CBetaScore: Statistical potential that evaluates pairwise interactions between Cβ atoms which are located within *cutoff* and that are at least *seq sep* residues apart. A score is assigned to each interaction using equally sized bins and distinguishing all possible pairs of proteinogenic amino acids.

ReducedScore: Statistical potential that evaluates pairwise interactions between the reduced representation of residues with Cα distance < *cutoff* and that are at least *seq sep* residues apart. Every residue gets represented by its Cα position *p* and a directional component *v* = *norm(ca_pos−n_pos)* + *norm(ca_pos − c_pos)*. For interacting residues *r1* and *r2*, we can define a line *l* between *p1* and *p2*. The statistical potential then considers:

- *dist*: distance between *p1* and *p2*
- *α*: angle between *v1* and *l*
- *β*: angle between *v2* and *l*
- *γ*: dihedral between *(p1 + v1, p1, p2, p2 + v2)*

A score is assigned to each combination of parameters using equally sized bins and distinguishing all possible pairs of proteinogenic amino acids.

HBondScore: Statistical potential that evaluates backbone hydrogen bonds as defined in the Rosetta energy function [1]. It considers the Cα, C and O positions from backbone hydrogen bond acceptors in interaction with the N and H positions from the backbone hydrogen bond donors. Four Parameters describe their relative orientation:

- *dist*: H-O distance
- *α*: O-H-N angle
- *β*: C-N-H angle
- *γ*: Cα-C-O-H dihedral angle

A scoring function with equally sized bins for all combinations of these parameters for three different states is generated. State 1 for helical residues, state 2 for extended residues and state 0 for all other residues. If the state of two interacting residues is the same, that is the one from which the score is extracted. In all other cases, the energy is extracted from the 0 state.

TorsionScore: Statistical potential that evaluates *φ/ψ* backbone dihedral angles taking into account the identity of the scored residue, but also its flanking residues. Instead of generating a scoring function with equally sized *φ* and *ψ* bins for all possible combinations of flanking residues (results in 20x20x20=8000 possible combinations), the flanking residues can be grouped arbitrarily. The default grouping scheme follows Solis & Rachovsky [2]. The first *φ* and last *ψ* angle of the scored loop are determined with the help of the scoring environment if set.

AllAtomInteractionScore: Statistical Potential that evaluates pairwise interactions between all heavy atoms that are located within *cutoff* and that are at least *seq sep* residues apart. A score is assigned to each interaction using equally sized bins and distinguishing all possible pairs of chemically distinguishable heavy atoms.

AllAtomPackingScore: Statistical potential that evaluates the number *N* of other heavy atoms within a *cutoff* around all heavy atoms of a residue not belonging to the assessed residue itself.
ClashScore: Calculates a simple clash score between all pairs of backbone atoms among the evaluated residues and towards the set environment. There is no need to define any parameters here as all interaction energies are fixed [3].
AllAtomClashScore: Same as ClashScore but considering all heavy atoms.
DensityScore: Given an input structure, the scorer generates a density map of the loop to be scored [4] and estimates the normalized cross correlation to a user defined target map.
SSAgreementScore: Evaluates a secondary structure agreement score as it is already defined for the SSAgreement Fragger score. In every score evaluation, the secondary structure of the loop is estimated by searching for hydrogen bonds leading to a secondary structure as defined by DSSP. The hydrogen bonds are searched internally in the loop as well as towards the environment.
PairwiseScore: Evaluates a list of generic pairwise functions. They are user defined and can either be simple contact functions (*x if dist <= max_dist, 0.0 otherwise*) or arbitrary discrete lookup tables. In case of lookup tables, the returned scores are linearly interpolated.

References

1. Kortemme T, Morozov AV, Baker D. An Orientation-dependent Hydrogen Bonding Potential Improves Prediction of Specificity and Structure for Proteins and Protein–Protein Complexes [Internet]. Journal of Molecular Biology. 2003. pp. 1239–1259. doi:10.1016/s0022-2836(03)00021-4

2. Solis AD, Rackovsky S. Improvement of statistical potentials and threading score functions using information maximization. Proteins. 2006;62: 892–908.

3. Canutescu AA, Shelenkov AA, Dunbrack RL Jr. A graph-theory algorithm for rapid protein side-chain prediction. Protein Sci. 2003;12: 2001–2014.

4. DiMaio F, Tyka MD, Baker ML, Chiu W, Baker D. Refinement of protein structures into low-resolution density maps using rosetta. J Mol Biol. 2009;392: 181–190.
