# Supplementary material for: ProMod3—A versatile homology modelling toolbox
Source: PLoS Comput Biol. 2021 Jan 28;17(1):e1008667. doi: 10.1371/journal.pcbi.1008667 (PMC7872268; doi:10.1371/journal.pcbi.1008667)
Supplement: S3 Text — (PDF) [file pcbi.1008667.s008.pdf]

Linear weights for loop candidate selection

To obtain a set of linear weights for any possible combination of scores, a large training setup has been generated. 4’000 chains have been randomly selected from the default structural database. 50’000 loops have been randomly chosen from those 4’000 chains with an equal distribution of 5’000 loops for each loop length in the range [3, 12] and the requirement of being no terminal loops and having more than 50% of the residues assigned as coil by DSSP. The 4’000 chains have been removed from the default structural database and a new fragment database has been built to query for non redundant candidates for all target loops. A total of 8’185’642 candidates have been found and fitted onto their corresponding stem residues with CCD.

For any combination of scores, the loop selection would choose a candidate with the best score for each loop in the training set. In order to find optimal linear weights to combine the scores, we maximize the probability of choosing a candidate which is similarly accurate as the best possible candidate. The motivation to compare with the best possible candidate instead of the correct solution is given by the large variation of observed accuracies in loop selection among different loop lengths. We have hence maximized the following target function:

$$\int_0^3 P[C\alpha RMSD_{chosen} - C\alpha RMSD_{best} \leq x \text{ Å}]dx \, ,$$

where Cα-RMSD (in Å) has been used to compare the chosen/best candidate with the correct solution. The resulting optimization problem turned out to be rather complex and a simple conjugate gradient approach showed poor convergence behaviour. CMA [1] as an alternative optimization strategy significantly improved the situation and allowed us to estimate weights for different score combinations.

References

1. Collette Y, Hansen N, Pujol G, Aponte DS, Le Riche R. Object-Oriented Programming of Optimizers - Examples in Scilab. Multidisciplinary Design Optimization in Computational Mechanics. 2013. pp. 499–538. doi:10.1002/9781118600153.ch14
