# Supplementary material for: ProMod3—A versatile homology modelling toolbox
Source: PLoS Comput Biol. 2021 Jan 28;17(1):e1008667. doi: 10.1371/journal.pcbi.1008667 (PMC7872268; doi:10.1371/journal.pcbi.1008667)
Supplement: S4 Text — (PDF) [file pcbi.1008667.s009.pdf]

Speed benchmarks

All data and scripts required to run the speed benchmarks are available at https://git.scicore.unibas.ch/schwede/promod3_pipeline_benchmark.
For ProMod3 specific benchmarks we used a Singularity container built with instructions from the documentation (ProMod3, version 3.1.0). Scripts calling external tools have been executed natively on a Linux system. Used Hardware:

- CPU: Intel i7-6600U 2.60GHz
- Memory: 16GB DDR4 2133 MHz
- HD: Dell NVMe LITEON 256GB SSD

Sidechain modelling speed benchmark: ProMod3 is compared to the binary of SCWRL4 as distributed by the Dunbrack lab. The corresponding scripts in the benchmark repository are *sidechain/reconstruct_sidechains_promod.py* and *sidechain/reconstruct_sidechains_scwrl.py*. Computation time is the average over three independent runs on the full test set used to measure sidechain modelling accuracy. The observed timings are:

- ProMod3 with subrotamers (FRM) and post-processing: 549 s
- ProMod3 no subrotamers (RRM): 253 s
- SCWRL4 with subrotamers (FRM): 1670 s
- SCWRL4 no subrotamers (RRM): 548 s

This gives the reported speedups of 3.0x in case of FRM and 2.2x in case of RRM.

Homology modelling speed benchmark: ProMod3 is compared to MODELLER (version 9.24) installed from the RPM package available from https://salilab.org/modeller/. The corresponding scripts in the benchmark repository are *modelling/build_promod_models.py* and *modelling/build_modeller_models.py*. Computation time is the average over three independent runs on the full test set used to measure homology modelling accuracy. The observed timings are:

- ProMod3: 1626 s
- MODELLER default settings: 2070 s

This gives the reported speedup of 1.3x.
